# Impact of Elective Caesarean Delivery on Double Burden of Malnutrition and Its Contribution to Wealth‐Based Inequality: A Decomposition Analysis Across South Asian Countries

**DOI:** 10.1002/puh2.70228

**Published:** 2026-04-09

**Authors:** Maliha Mahazabin, Khondokar Naymul Islam, Md. Alamgir Sarder

**Affiliations:** ^1^ Statistics Discipline, Science Engineering and Technology School, Khulna University Khulna Bangladesh

**Keywords:** decomposition, delivery mode, double burden of malnutrition, elective caesarean section, inequalities, South Asia, undernourished child

## Abstract

**Background:**

The double burden of malnutrition at household level (DBMHL), which denotes the coexistence of undernutrition and overweight or obesity within the same household, underscores the complex and multifaceted nature of malnutrition. DBMHL is a critical issue in South Asia (SA) and mostly influenced by dietary transitions and healthcare access disparities as well as intricately linked to socioeconomic inequality. The primary aim of this research is to determine the impact and contribution of elective C‐section to the wealth‐based inequality in DBMHL.

**Methods:**

Participants (208,822) were taken from the most recent Demographic and Health Survey (DHS) datasets for the analysis. We applied bivariate statistics, binary logistic regression, concentration curve, concentration index (CIX), and decomposition analysis to determine the factors and socioeconomic inequalities related to DBMHL in the selected SA countries.

**Results:**

The overall prevalence of DBMHL was 7.80% in the selected SA countries, and the extent of disparity exists much among affluent groups (CIX: 0.217; *p* < 0.001). The decomposition analysis revealed that elective C‐section delivery contributed 10.91% to the pro‐rich socioeconomic inequality in DBMHL. Mothers who opted for elective caesarean sections (CSs) (adjusted odds ratios [AOR]: 1.77; 95% CI: 1.63–1.91) were more likely to report DBMHL than mothers who opted for normal vaginal delivery.

**Conclusion:**

Our findings suggest elective cesarean section is an important contributor to wealth‐related inequality in DBMHL, strengthening clinical governance and counseling around nonmedically indicated elective CS may help to improve maternal and child health outcomes in SA. Public health practitioners and policymakers should promote awareness among women of reproductive age through mass media regarding healthy weight management before pregnancy and the potential implications of elective CS for the overall well‐being of mother–child dyads.

## Graphical abstract

Double burden of malnutrition at household level (DBMHL) is a new challenging public health concern in South Asian countries, defined by the coexistence of overweight mothers and undernourished children. With 208,822 respondents’ information, the decomposition analysis revealed that DBMHL was more prevalent in the wealthiest families, and elective cesarean section is one of the key contributor to DBMHL (contributing 10.91% to the pro‐rich socioeconomic inequality in South Asia).

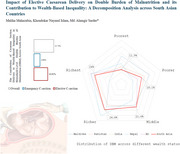

## 1 Introduction

Double burden of malnutrition (DBM) is a new challenging public health concern in developing countries defined by the coexistence of overweight mothers and undernourished children at the household level [1]. Overweight and obesity in women raise the risk of maternal and neonatal morbidity, whereas undernutrition accounts for 3.1 million of all child fatalities [2, 3]. In 2022 millions of children worldwide struggled with undernutrition, with 149 million being stunted (being shorter than usual for their age), 45 million being wasted (weight below than usual for their height), and 82 million being underweight (weight below than usual for their age) [4, 5]. With an emphasis on well‐being and improved nutrition, the Sustainable Development Goals (SDGs) focus on ending all forms of malnutrition and improving access to nutritious food, particularly among children [6]. Notably, target 2.2 of SDG aims to end all forms of malnutrition by 2030, including stunting and wasting in children under 5, and addressing the nutritional needs of women and older persons, emphasizing the urgency of identifying the risk factors of malnutrition and taking action to mitigate its effects [7].

Since 1980, maternal obesity has been rising steadily, primarily due to rapid urbanization, shifting dietary patterns, and increasingly sedentary lifestyles [3, 8]. Assuming that overweight individuals have adequate micronutrients and do not experience hidden hunger (micronutrient deficiency despite adequate calorie intake) is not valid. One prevalent manifestation of hidden hunger worldwide is the inadequate iron levels observed in women [9, 10]. Reducing the nutritional value of one's diet typically leads to overconsumption of energy‐dense meals rich in carbs and fats but lacking in essential nutrients, which may potentially lead to obesity [11]. Undernutrition during childhood heightens the chance of obesity in adulthood, especially when individuals encounter an obesogenic environment [12]. Therefore, the DBM in the mother–child dyad is not paradoxical. A woman who is overweight may have experienced inadequate nourishment during her childhood. Experiencing malnutrition throughout the early stages of life, followed by subsequent obesity, elevates the susceptibility to noncommunicable diseases over the lifespan, as well as heightens the likelihood of complications during childbirth in women [5, 13].

Undernutrition has a detrimental effect on mineral density and bone formation of children [14]. The metabolism and health of an individual in adulthood are significantly influenced by their food habits during the early stages of life [15]. The function of the gut microbiome and insulin signaling can be disrupted due to the long‐term effects of malnutrition in early life [13]. The mode of delivery, particularly planned or elective caesarean section (CS), can alter the composition and stability of the infant gut microbiota and feeding practices in ways that may affect infant nutritional status [16, 17, 18]. Elective CS is frequently associated with delayed initiation and lower rates of exclusive breastfeeding, reducing the early transfer of protective nutrients and beneficial microbes needed for growth and immunity [19, 20]. In comparison to healthy children, malnourished children exhibit an imbalance in their gut microbiota [12, 21], which leads to a variety of morbidities, including autoimmune disease, cancer, cardiovascular disease, and diabetes later [22]. Furthermore, studies have shown that being underweight, wasting, and stunting are responsible for 19.6%, 14.6%, and 14.5% of fatalities and 18.7%, 14.8%, and 12.6% of disability‐adjusted life‐years (DALY) loss among under‐five children, respectively [23].

Asia has one of the highest proportions of malnutrition cases, with 54% of children experiencing stunted growth and 69% experiencing wasting, particularly in southern Asia; the child stunting rate (31.7%) is considerably high compared to other regions [24]. However, due to demographic shifts and economic growth over the past few decades, the South and Southeast Asia region has consistently seen a decline in child undernutrition while simultaneously reporting an increase in overweight or obesity among women [1]. DBM in the mother–child dyad has been rising steadily in South Asian (SA) countries, driven by urbanization, economic growth, and shifts in dietary patterns [25]. The number of cases of overweight among women in Bangladesh raised from 11.4% to 25.2% between 2004 and 2014 [8]. The unequal distribution of DBM across socioeconomic or wealth‐based groups is an important public health concern in SA. Previous researchers have demonstrated that respondents with higher incomes or educational attainment were less likely to have underweight children in SA countries [26, 27]. Moreover, maternal underweight and overweight greatly raise the risk of undernutrition in children [3]. Research on childhood malnutrition in Pakistan identified the body mass index (BMI) as a major contributor to the reported disparity in socioeconomic inequality [28]. Another research conducted in Bangladesh reveals that the prevalence of DBM almost tripled between 2007 and 2017, and children who were delivered by CS are more vulnerable to DBM [29]. In India, economically affluent individuals have a higher prevalence of DBM. Moreover, the level of education attained by mothers and the method of delivery, particularly CS, are key factors contributing to the observed disparity in socioeconomic inequality in DBM [30].

Prior research has primarily examined either overnutrition or undernutrition, yet no study has directly targeted the coexistence of the problems at the household level and its association with elective CS in SA countries. To accomplish the fundamental goal of the SDGs, it is essential to determine the current scenario of double burden of malnutrition at the household level (DBMHL). Therefore, this study focuses on wealth‐based discrepancy of DBMHL in SA. As well as previous studies have not yet explored the socioeconomic disparity of DBMHL within this specific area (SA). The primary objective of this research is to determine the association between elective CS and the co‐occurrence of overweight/obesity among mother and stunting/wasting/underweight among children from the same household. Another aim is to observe the contribution of elective CS to the wealth‐based inequality in household‐level DBM after adjusting potential covariates. The main hypothesis of our study is that the type of delivery, especially elective cesarean section, is linked to the occurrence of DBMHL among mother–child pairs. The insights obtained from this research might assist policymakers in establishing strategies for addressing and diminishing the inequality of DBM in developing countries.

## 2 Materials and Methods

### 2.1 Data Source

This study utilized the Demographic and Health Survey (DHS) datasets from SA countries, including only those with complete information on the timing of the decision regarding delivery type (elective or planned and emergency or unplanned). We selected only the most recent dataset for each country, with surveys conducted within the past 10 years, including Bangladesh (BDHS 2017–18), Pakistan (PDHS 2017–18), India (IDHS 2019–21), the Maldives (2016–17), and Nepal (2016). The DHS are nationally representative surveys conducted in compliance with national regulations that utilize a multistage, stratified sample technique aimed at collecting reliable information on health and nutrition for women, children, and households. Ever‐married mothers with children were approached regarding their children's nutritional status. The DHS followed the World Health Organization's (WHO) recommendations for determining the cut‐off values for mothers’ BMI (i.e., overweight, normal, and underweight) and children's underweight, wasting, or stunting status. Wasting, underweight, and stunting were defined for a child based on *Z*‐scores below the 2‐standard deviation for weight‐for‐height (WHZ), weight‐for‐age (WAZ), and height‐for‐age (HAZ) *Z*‐scores, respectively. To achieve our research aim, we considered the sociodemographic, maternal pregnancy, and child‐related data based on the literature and accessibility. The initial data refinement step focused on selecting data from nonpregnant mothers with at least one living child under the age of 5. In the second step, we omitted any incomplete or unreported responses for both the considered outcome and explanatory variables. This process yielded a dataset comprising information from 208,822 mother–child dyads.

### 2.2 Outcome Variable

The WHO defines the DBM as the simultaneous presence of undernutrition alongside overweight, obesity, or diet‐related noncommunicable diseases that occur within the population, households, and individuals throughout different stages of life [31]. As undernourished children are more prevalent than overweight children in SA countries [26], we considered overweight mothers and undernourished children to determine the DBMHL. The outcome variable (DBMHL) was coded “1” if the mother was overweight and had at least one child under 5 in the household who was undernourished (stunting/underweight/wasting) and coded “0” if otherwise.

### 2.3 Explanatory Variable

The primary explanatory variable is the elective C‐section delivery in the most recent pregnancy. In the DHS datasets from SA countries, information on CS type was based on maternal self‐report. Women were asked whether the childbirth was by CS and, if so, whether the decision was made before or after labor began. C‐sections decided before labor were considered elective or planned, and those after labor onset as emergency or unplanned [32]. We classified the variable mode of delivery as “emergency CS,” “elective CS,” and “normal vaginal delivery (NVD).” An emergency CS was performed due to severe circumstances during childbirth, whereas an elective CS was a personal decision for the procedure.

### 2.4 Potential Covariates

We divided our study covariates into three groups as follows: sociodemographic, maternal pregnancy‐related, and child factors. Sociodemographic factors included “place of residence,” “country,” “advanced maternal age (≥35),” “maternal education,” “household wealth status,” and “frequency of watching television.” Factor related to maternal pregnancy included “ever had pregnancy termination.” Child factors included “child age (in months)” and “maternal parity (≤2, >2).”

### 2.5 Statistical Analysis

**FIGURE 1:**
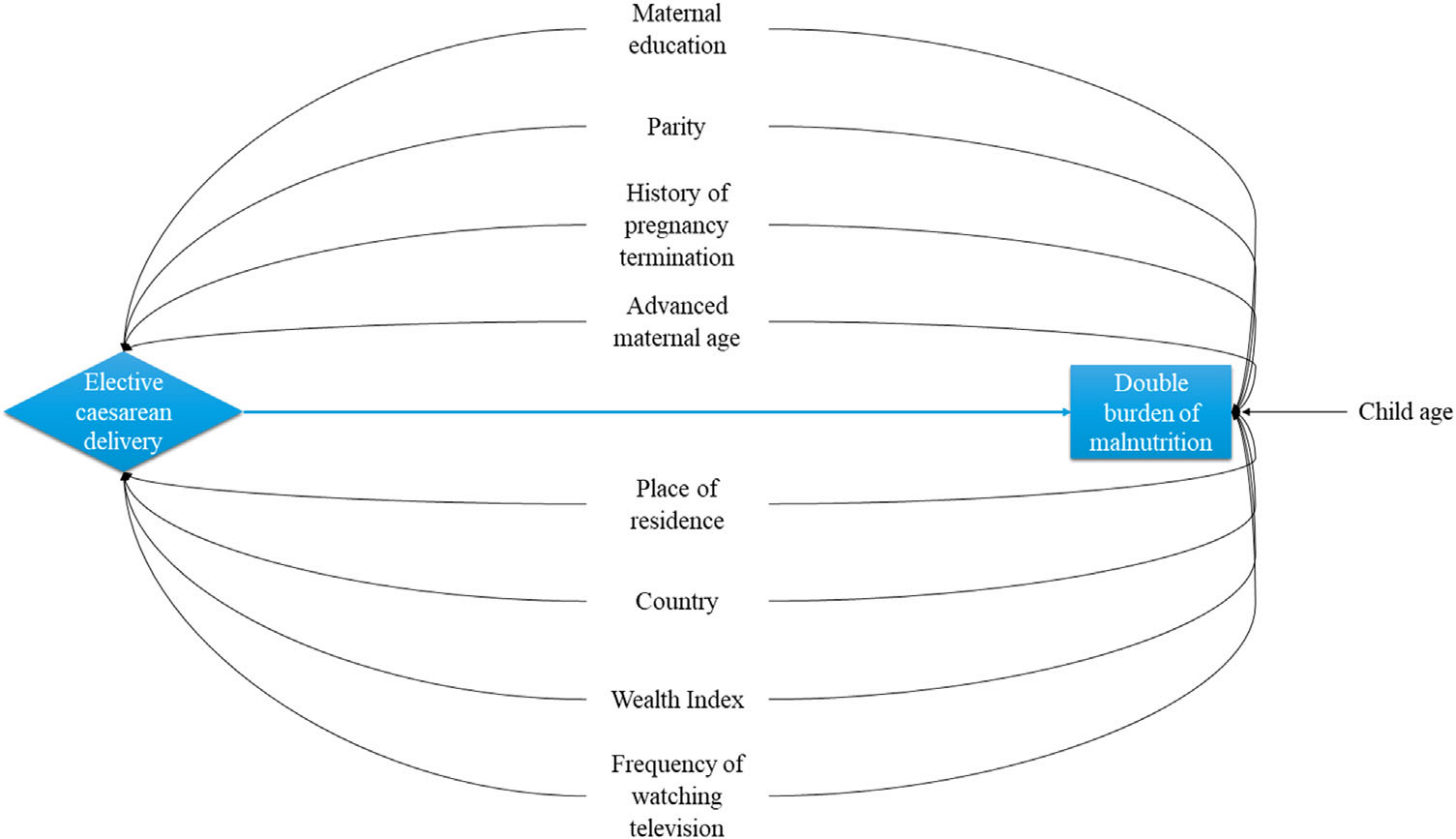
Directed acyclic graph (DAG) demonstrating the conceptual framework.

All our selected potential covariates based on literature and available data in hand may play a confounding role in our analysis except for the child age. Child age would act as a predictor of the outcome but not a confounder. Factors like infant feeding practices (early breastfeeding initiation, exclusive breastfeeding, and infant and young child feeding practices), gut microbiota, and maternal postpartum weight retention were not controlled, as they might have a mediating effect on the relationship between cesarean delivery and the DBM. Figure 1 illustrates our conceptual framework using a directed acyclic graph (DAG). In order to ensure the statistical reliability of our analyses, we incorporated sample weights, clustering, and stratification weights following the DHS guideline. The characteristics of the data and the distribution of DBMHL among the explanatory variables are tabulated using descriptive statistics. Then, we performed both unadjusted and adjusted binary logistic regression models to analyze the pooled data of considered selected SA countries, which resulted in effect sizes with crude odds ratios (COR), adjusted odds ratios (AOR), and marginal effects, respectively, for a 5% level of significance. Stata 17 (MP) software was used for the statistical analysis. The area under the curve (AUC) value for the fitted logistic model was 0.65 (using “predict” and “roctab” commands), demonstrating a modest classification of the outcome, and no multicollinearity was detected among factors (variance inflation factor [VIF] <2.5). The multicollinearity among exposure variables was checked using the “vif” command, constructing the same model with the “regress” command adjusting for sampling weight. Obtained VIF values are provided in Table S3. The inequality of DBMHL based on the poorest to richest groups of household wealth index was assessed using the Wagstaff concentration index (CIX) [33]. The concentration curve (CC) was used to visualize the overall and country wise wealth‐based inequalities of DBMHL [34]. The user‐written Stata command “conindex” was used to determine the CIX [35].

## 3 Results

**FIGURE 2:**
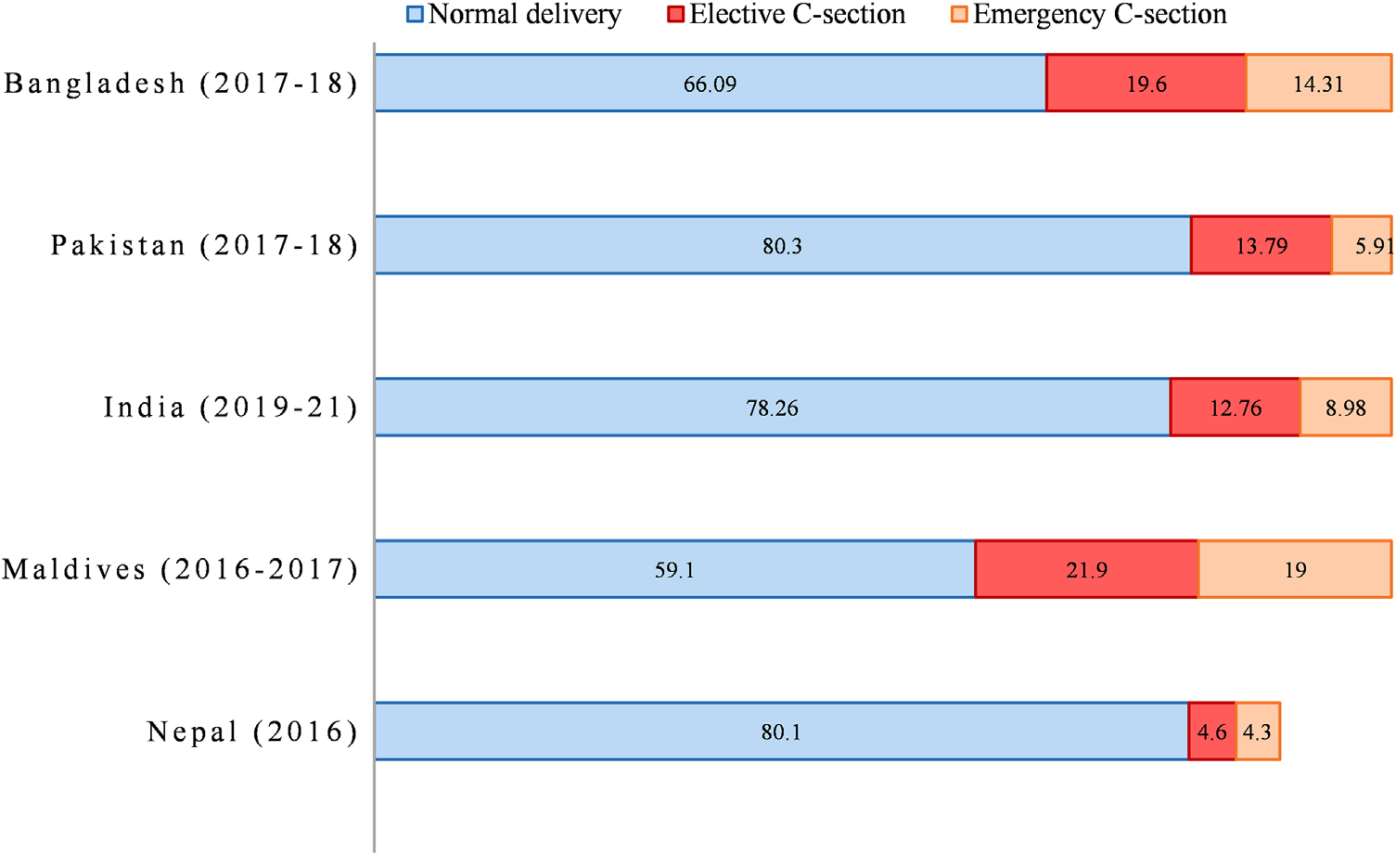
Mode of delivery by country.

A total of 208,822 respondents were included in this study, where the largest share was from India (93.7%), then from Bangladesh (2.3%), and the rest came from Pakistan, Maldives, and Nepal. Overall, DBMHL in SA was found 7.8% in this study, and this percentage in Bangladesh, Pakistan, Maldives, Nepal, and India was 6.46, 16.11, 12.1, 4.7, and 7.18, respectively (Figure S1). Table 1 represents the distribution of the pooled data where 78.6% of the participants were from rural areas. Approximately 21.5% of the women had no formal education, 46.2% of women watch television at least once a week, and 23.84% were from the poorest households. The study also found that the majority of women had opted for the NVD (77.6%). Delivery by elective CS was higher in Bangladesh (19.6%) than in Pakistan and India (Figure 2). Approximately 15.1% of women had a terminated pregnancy, and 13.0% chose elective CS delivery. In Table S1, we showed the crude and AOR resulted in our multivariable logistic regression models performed on the aggregated data of considered SA countries, and findings revealed that DBMHL was 33% more frequent among urban respondents than rural ones (AOR: 1.33; 95% CI: 1.24–1.41), mothers who ever had a terminated pregnancy were 9% more likely (AOR: 1.09; 95% CI: 1.02–1.17) times more likely to report DBMHL, and emergency CS delivery showed 1.59 (95% CI: 1.47–1.72) times higher likelihood of reporting DBMHL.

**TABLE 1:** Pooled weighted background characteristics of the study participants across countries.

		Frequency (*N*)	Percentage
**Outcome variable**			
Double burden of malnutrition	No	193,604	92.2
	Yes	15,218	7.8
**Explanatory variable**			
Mode of delivery	NVD	166,949	77.6
	Emergency CS	17,563	9.4
	Elective CS	24,310	13.0
**Sociodemographic factors**			
Place of residence	Urban	44,621	21.4
	Rural	164,201	78.6
Advanced maternal age	No (<35 years)	187,105	89.6
	Yes (≥35 years)	21,717	10.4
Maternal education	No education	44,889	21.5
	Primary	27,658	13.2
	Secondary	106,868	51.2
	Higher	29,407	14.1
Wealth index	Poorest	55,282	26.5
	Poorer	48,722	23.3
	Middle	40,984	19.6
	Richer	35,584	17.1
	Richest	28,250	13.5
Frequency of watching television	Not at all	68,564	32.9
	Less than once a week	43,773	20.9
	At least once a week	96,485	46.2
Country	Bangladesh	4810	2.3
	India	195,740	93.7
	Pakistan	3701	1.8
	Maldives	2343	1.1
	Nepal	2228	1.1
**Maternal pregnancy‐related factors**			
Ever had terminated pregnancy	No	177,378	84.9
	Yes	31,444	15.1
**Child‐related factors**			
Child age (in months)	≤24	89,269	42.7
	>24	119,553	57.3
Maternal parity	≤2	137,488	65.8
	>2	71,334	34.2

Abbreviations: CS, caesarean section; NVD, normal vaginal delivery.

Table 2 highlights the marginal effect and AOR of elective C‐section with DBMHL by pooled and individual SA countries after controlling the potential covariates of the study. Mothers who had elective C‐section (AOR: 1.77; 95% CI: 1.63–1.91) were more likely to experience DBMHL than mothers who had NVD. Moreover, the significant association between elective C‐section with DBMHL was found among respondents from Bangladesh (AOR: 1.45; 95% CI: 1.04–2.05) and India (AOR: 1.81; 95% CI: 1.67–1.96).

**TABLE 2:** Findings from binary logistic regression to identify association between household‐level double burden of malnutrition and elective C‐section for each country and overall South Asia.

Country/Region	Crude odds ratio (95% CI)	Adjusted odds ratio^a^ (95% CI)	Marginal effect^a^ (95% CI)
South Asia	2.14 (1.99–2.30)^*^	1.77 (1.63–1.91)^*^	0.040 (0.034–0.045)^*^
Bangladesh	1.69 (1.24–2.30)^*^	1.45 (1.04–2.05)^*^	0.023 (0.002–0.043)^*^
Pakistan	1.20 (0.81–1.77)	1.14 (0.75–1.74)	0.016 (−0.035 to 0.067)
India	2.20 (2.04–2.37)^*^	1.81 (1.67–1.96)^*^	0.041 (0.035–0.047)^*^
Maldives	1.03 (0.72–1.48)	1.05 (0.73–1.52)	0.005 (−0.032 to 0.043)
Nepal	2.27 (0.92–5.60)	1.32 (0.33–2.60)	0.011 (−0.032 to 0.055)

Abbreviation: CI, confidence interval.

^a^Estimates adjusted for sociodemographic, maternal pregnancy, and child‐related factors.

*p < 0.05.

**FIGURE 3:**
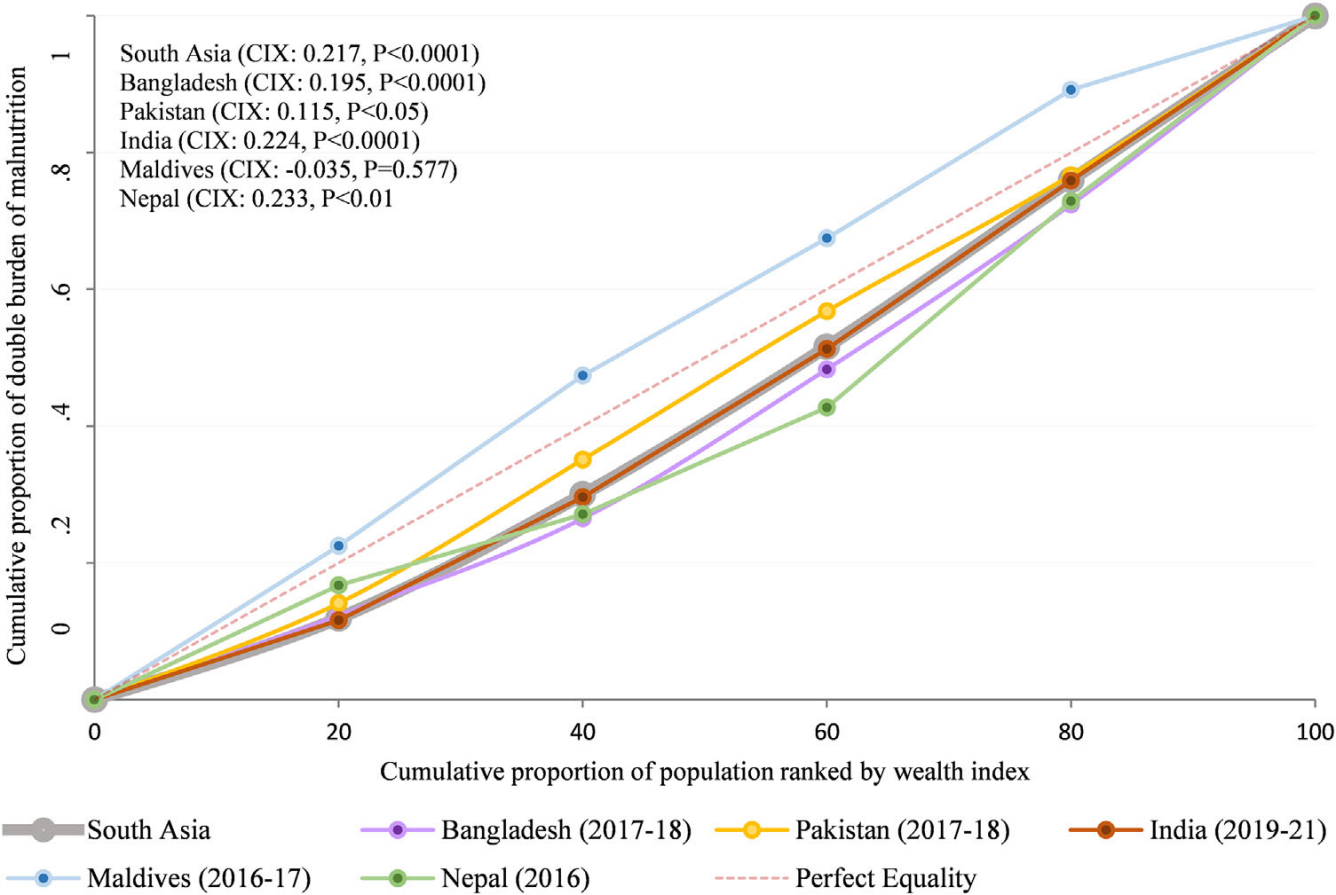
Concentration curve for wealth‐based inequality in double burden of malnutrition. CIX, concentration index.

Figure 3 depicts DBMHL's CC in five SA countries, both individually and as a whole. The curves are below the line of equality (45‐degree line), which indicates that for each of three countries and the overall SA region, the DBMHL is concentrated among the wealthiest households. Nepal shows the highest CIX (0.233, *p* < 0.01), which means that DBMHL is most concentrated among wealthier households there, followed by India (0.224, *p* < 0.0001), Bangladesh (0.195, *p* < 0.0001), and Pakistan (0.115, *p* < 0.05).

**FIGURE 4:**
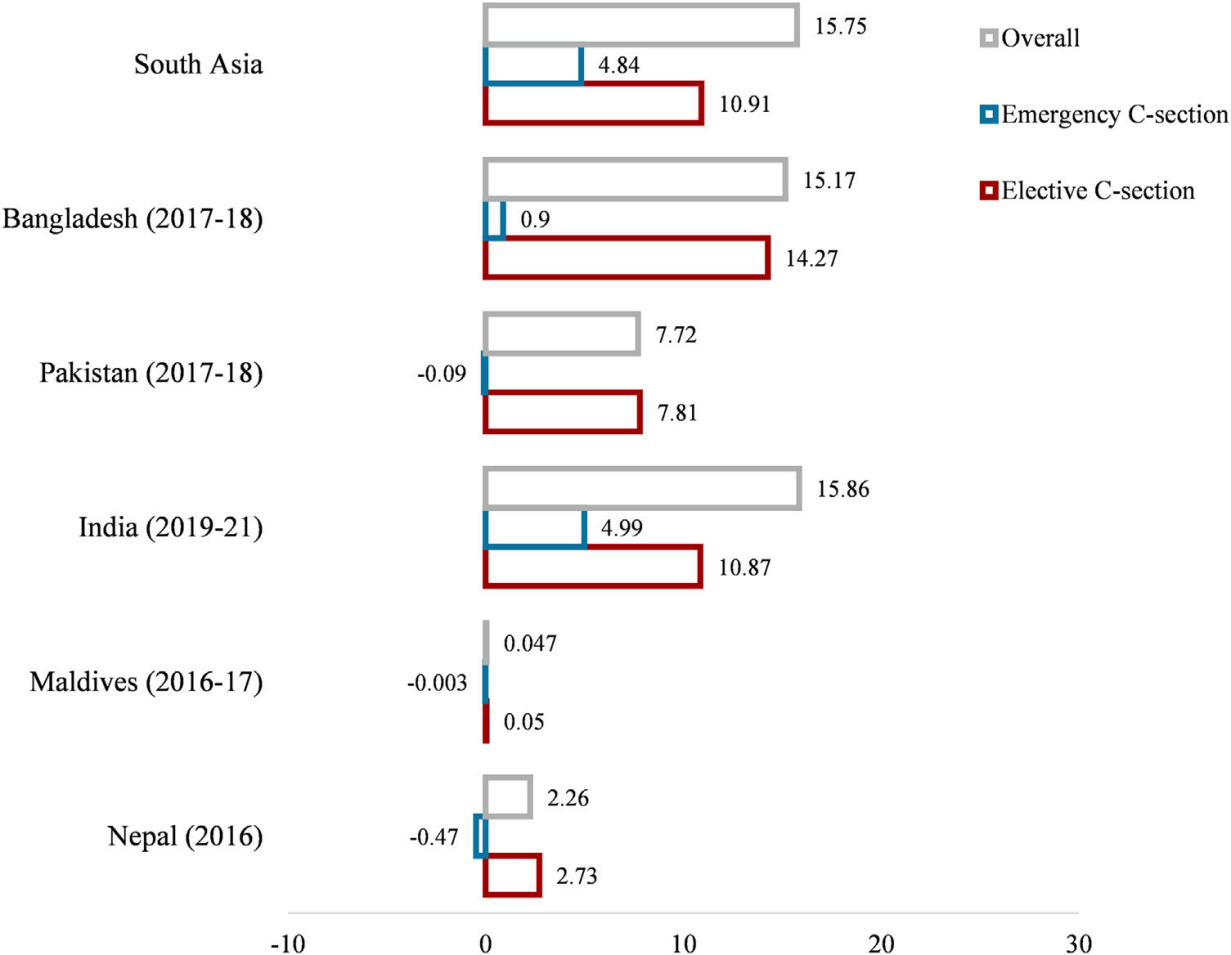
Percentage contribution of C‐section delivery to the corresponding country‐ or region‐specific wealth‐based inequality in the double burden of malnutrition.

In this study, we examined inequality using the wealth index and obtained the Wagstaff CIX value. The CIX (0.217) for DBMHL was positive (pro‐rich), with *p* < 0.001. This indicates that DBMHL was more prevalent in the wealthiest families, providing evidence in favor of our CC. A decomposition analysis was also conducted on the basis of the Wagstaff method (the full set of results is reported in Table S2). The percentage contribution shows how much each predictor adds to overall socioeconomic differences in DBMHL. A positive sign indicates a contributing variable increases the observed inequality, whereas a negative sign indicates that a contributing variable results in a decrease in the observed inequality. Figure 4 presents the selected SA countrie's decomposition analysis, where the *y*‐axis indicates the percentage contribution to the inequality in DBMHL on delivery mode. In SA, the mode of delivery contributed positively (15.75%) to the DBMHL, where 10.91% was for elective C‐section and 4.84% was for emergency C‐section. Regarding elective C‐section, the positive percentage contribution for the pro‐rich socioeconomic inequality in DBMHL was highest in Bangladesh (14.27%), subsequently India (10.87%) and Pakistan (7.81%). The lowest positive percentage contribution of DBMHL on elective C‐section was observed in Maldives (0.05%), followed by Nepal (2.73%).

## 4 Discussion

DBM among mother–child dyads is a serious concern in selected SA countries (Bangladesh, Pakistan, Maldives, Nepal, and India). This study determined the socioeconomic inequality and association between elective C‐section with the coexistence of overnutrition among mothers and undernutrition among children in the same household and found a substantial socioeconomic gap of DBMHL in the selected SA countries. Our study revealed that the prevalence of DBMHL (pooled of five countries) is 7.80%, which was higher than the prevalence rate of DBMHL in African countries, such as Ethiopia (3.6%) [36] and rural Kenya (3%) [37]. Our study found that the undernutrition among Indian children (33.59% stunted, 19.78% wasted, and 28.11% underweight) is relatively higher than that of children in Bangladesh (30.13% stunted, 8.22% wasted, and 19.32% underweight) and Pakistan (37.44% stunted, 7.96% wasted, and 21.02% underweight), which aligns with prior studies [28, 29, 30, 38]. The prevalence of DBMHL is higher in Pakistan (16.11%) than in Bangladesh (6.46%) and India (7.18%). We found that almost half of the women of reproductive age in Pakistan are overweight or obese. The rate of overweight women in Maldives and Pakistan is alarmingly higher than in other SA countries, such as Bangladesh, Nepal, and India, which is in line with a previous study report [38]. An increment in the prevalence of obesity among women in SA is linked to poor sleeping patterns, early‐life malnutrition, the transition to modern lifestyle choices, and the reduction of physical activities [39].

The decomposition analysis of this study determined that elective C‐section delivery contributed to the pro‐rich socioeconomic inequality in DBMHL. Previous studies reported that CS delivery is associated with DBMHL in SA countries [29, 30]; however, no studies revealed whether elective CS or emergency CS has a greater impact on higher concentrations of DBMHL in the wealthiest groups. Our study revealed substantial contribution of DBMHL on elective CS than emergency CS for each SA country; particularly in Bangladesh, the percentage contribution of DBMHL on elective CS is the highest among SA countries. The NVD is the preferred method for the natural progression of pregnancy, whereas CS delivery should only be considered in emergencies or for pregnancies where it is necessary to ensure the safety of mother and child [40]. However, in many Asian countries, the CS delivery rate is increasing rapidly, which negatively influences the child's overall well‐being. Our study also revealed a considerable shift toward CS delivery, deviating from the WHO‐recommended ideal range of 10%–15% [41]. Earlier studies revealed that in SA, both private and some public hospitals reported high rates of elective CS, often without clear medical necessity [32, 42]. Contributing factors influencing this trend include hospital and physician financial incentives as well as the ability to schedule deliveries at convenient times [43, 44]. Elective CS can also be influenced by nonmedical factors, including fear of labor pain, perceived convenience, sociocultural norms, and concerns about physical appearance, as qualitative evidence indicated that some primiparous women viewed CS as preferable to avoid the structural changes to the pelvic and genital anatomy [45]. Prior studies in SA have shown that caesarean deliveries are more prevalent among women from affluent households and those with overweight, as excess weight can increase the likelihood of labor complications and prompt healthcare providers to recommend CS [32, 46]. Additionally, a systematic review showed that misconceptions about vaginal birth, prior obstetric experiences, maternal anxiety, and inadequate knowledge of potential complications drove many women to request elective CS [47]. This present study also revealed that mothers who opted for elective CS delivery were 77% more likely to experience DBMHL than mothers who opted for NVD. Notably, an elective CS poses serious long‐term consequences for both the mother and the infant. Infants delivered via elective CS lack contact with maternal microorganisms during birth, which increases their susceptibility to developing celiac disease [17, 19]. In addition, elective CS births result in a higher incidence of neonatal unit enrollment, breathing issues, anemia, and the requirement for oxygen support [16, 17]. Gut microbes are passed from mother to infant during and after birth, and in CS delivery, particularly elective CS, the lack of contact with maternal microorganisms during birth can negatively affect the gut microbiota composition of the child [18, 48]. Gut microbiota plays a crucial role in the overall health of human beings, as studies indicated that any disruption of gut microbiota (i.e., gut dysbiosis) can potentially have adverse effects on the child's development and raise the risk of chronic illnesses later in life [21, 22, 48, 49]. This disruption may interfere with nutrient absorption, energy metabolism, and immune system [49, 50], providing a biologically plausible explanation for the observed association between elective CS and child undernutrition. Infants born via elective CS often experience delayed initiation and shorter duration of exclusive breastfeeding, which limits the supply of essential nutrients needed for healthy growth and immune development [20]. These biological (gut microbiota) and behavioral (breastfeeding) disruptions may partly explain the higher odds of undernutrition among children delivered by elective CS, contributing to the observed DBM in mother–child pairs.

### 4.1 Strengths and Limitations

Our primary strength of this study was the utilization of nationally representative data from five SA countries (Bangladesh, Pakistan, India, Maldives, and Nepal) to determine the inequality of DBMHL. However, there are some limitations in the study. As the DHS was a cross‐sectional study, it was not possible to determine any causal relationship. The classification of elective and emergency CSs was based on maternal self‐report, which may be subject to recall or reporting bias. Furthermore, the DHS datasets either did not include the factors related to food intake (mothers’ diet patterns during pregnancy, knowledge about a healthy diet, and a child's daily food intake), or most cases were missing, making it not feasible to determine the association between DBMHL and the food intake pattern of mother–child pairs. India contributes a large portion of the pooled sample, which influences the pooled regression and CIX decomposition results. Therefore, results are presented for each country to address heterogeneity and reduce reliance on pooled estimates.

## 5 Conclusion

The coexistence of mother overweight and child undernutrition in Bangladesh, Pakistan, and India presents a significant challenge that demands urgent and comprehensive interventions. Mother–child pairs from the richest wealth quintiles were more vulnerable to the DBMHL in the selected SA countries.

The findings of this study suggest that while developing health policy, it is crucial to consider both the associated factor and the level of socioeconomic inequality in DBMHL. Elective CS delivery among women increases the socioeconomic disparity of DBMHL in the richest households, highlighting that women from affluent groups are more inclined to choose elective CS delivery even when the case is not an emergency. Public health practitioners should monitor and regulate nonmedically indicated elective C‐sections (particularly focusing on affluent groups who frequently opt for elective C‐sections without medical necessity), implement campaigns to raise awareness regarding the long‐term nutritional risks for children delivered through elective CS, and promote prepregnancy weight management programs to reduce the prevalence of DBMHL in SA countries. Policymakers may consider strengthening monitoring and regulatory mechanisms to address potential financial incentives associated with elective (unnecessary or planned) caesarean deliveries across SA countries [42, 43, 44]. Governments in these countries should establish and rigorously enforce national guidelines to limit elective C‐sections to medically necessary cases, in line with WHO recommendations, to reduce unnecessary procedures. Policymakers must target both public and private health workers to encourage women during their antenatal care visit to opt for an NVD rather than elective CS to mitigate socioeconomic inequality in DBMHL among mother–child dyads.

## Author Contributions

Maliha Mahazabin, Khondokar Naymul Islam, and Md. Alamgir Sarder conceptualize the research. Maliha Mahazabin and Khondokar Naymul Islam contributed data curation, designed the research study, and conducted formal analysis. Maliha Mahazabin wrote original draft. Maliha Mahazabin, Khondokar Naymul Islam, and Md. Alamgir Sarder contributed to writing – review and editing.

## Ethics Statement

The data were assessed through the DHS program (http://www.dhsprogram.com).

## Consent

The DHS has strict requirements for obtaining participants’ informed consent and confidentiality of the identities.

## Conflicts of Interest

The authors declare no conflicts of interest.

## Supporting information




**Supporting File 1**: puh270228‐sup‐0001‐SuppMat.pdf

## Data Availability

The data that support the findings of this study are openly available in DHS at http://www.dhsprogram.com.
