# Supplementary material for: Impact of Elective Caesarean Delivery on Double Burden of Malnutrition and Its Contribution to Wealth‐Based Inequality: A Decomposition Analysis Across South Asian Countries
Source: Public Health Chall. 2026 Apr 9;5(2):e70228. doi: 10.1002/puh2.70228 (PMC13063393; doi:10.1002/puh2.70228)
Supplement: Supplementary file 1 — Supporting File 1: puh270228‐sup‐0001‐SuppMat.pdf [file PUH2-5-e70228-s001.pdf]

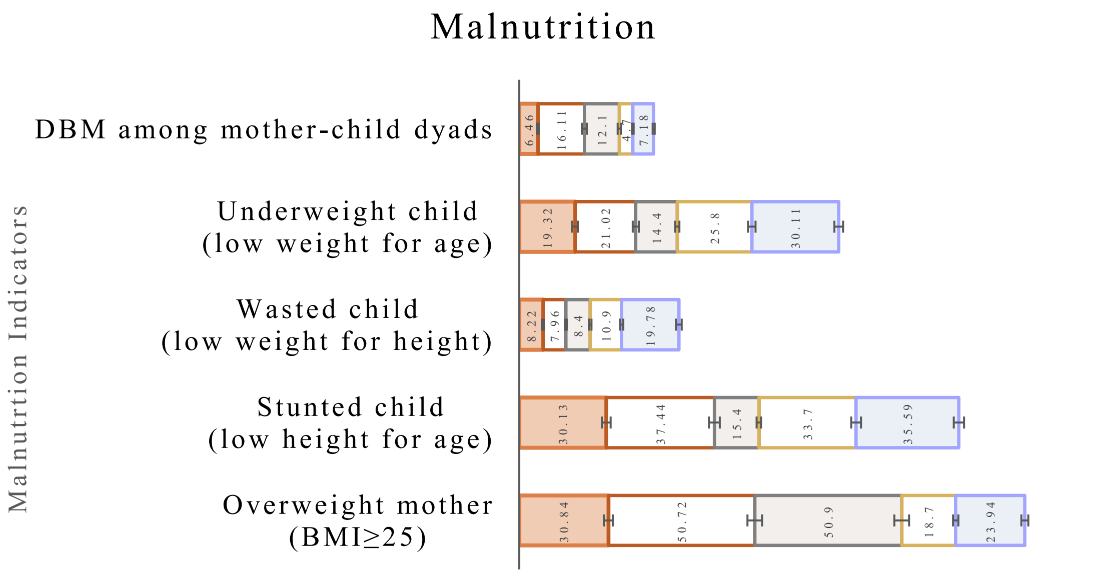


| | Overweight mother (BMI≥25) | Stunted child (low height for age) | Wasted child (low weight for height) | Underweigh t child (low weight for age) | DBM among mother-child dyads |
|---|---|---|---|---|---|
| Bangladesh (2017-18) | 30.84 | 30.13 | 8.22 | 19.32 | 6.46 |
| Pakistan (2017-18) | 50.72 | 37.44 | 7.96 | 21.02 | 16.11 |
| Maldives (2016-17) | 50.9 | 15.4 | 8.4 | 14.4 | 12.1 |
| Nepal (2016) | 18.7 | 33.7 | 10.9 | 25.8 | 4.7 |
| India (2019-21) | 23.94 | 35.59 | 19.78 | 30.11 | 7.18 |

**Figure S1**. Prevalence of malnutrition indicators by country

**Table S1.** Binary logistic regression to identify factors associated with double burden of malnutrition

| **Variables** | | **COR (95% CI)** | **AOR (95% CI)** | **Marginal effect** |
|---|---|---|---|---|
| **Primary explanatory factor** | | | | |
| Mode of delivery | Normal vaginal delivery (ref) | | | |
| | Emergency C-section | 1.82 (1.68-1.96)* | 1.59 (1.47- 1.72)* | 0.032 |
| | Elective C-section | 2.14 (1.99-2.30)* | 1.77 (1.63-1.91)* | 0.040 |
| **Socio-demographic factors** | | | | |
| Place of residence | Rural (ref) | | | |
| | Urban | 1.85 (1.75-1.96)* | 1.33 (1.24-1.41)* | 0.020 |
| Advanced maternal age | No (< 35 years) (ref) | | | |
| | Yes (≥ 35 years) | 1.72 (1.59-1.86)* | 1.53 (1.41-1.67)* | 0.030 |
| Maternal education | No education(ref) | | | |
| | Primary | 1.20 (1.09- 1.33) | 1.08 (0.98-1.20) | 0.005 |
| | Secondary | 1.43 (1.33- 1.53) | 1.10 (1.01-1.19)* | 0.006 |
| | Higher | 1.79 (1.64- 1.95) | 1.03 (0.93-1.14) | 0.002 |
| Wealth Index | Poorest(ref) | | | |
| | Poorer | 1.71 (1.57-1.87)* | 1.62 (1.48-1.78)* | 0.034 |
| | Middle | 2.30 (2.11-2.51)* | 1.99 (1.82-2.19)* | 0.048 |
| | Richer | 2.78 (2.55-3.04)* | 2.21 (1.99-2.45)* | 0.055 |
| | Richest | 3.30 (3.02-3.60)* | 2.33 (2.08-2.62)* | 0.059 |
| Frequency of watching television | Not at all(ref) | | | |
| | Less than once a week | 1.38 (1.27-1.49)* | 1.04 (0.95-1.14) | 0.003 |
| | At least once a week | 1.76 (1.65-1.88)* | 1.10 (1.02-1.19)* | 0.007 |
| Country | India (ref) | | | |
| | Pakistan | 2.04 (1.73- 2.40)* | 1.07 (1.47- 2.07)* | 0.039 |
| | Bangladesh | 0.84 (0.73-0.96)* | 0.82 (0.71-0.94)* | -0.010 |
| | Maldives | 1.69 (1.39-2.05)* | 1.34 (1.09-1.66)* | 0.020 |
| | Nepal | 0.55 (0.42-0.72)* | 0.55 (0.42-0.72)* | -0.040 |
| **Maternal pregnancy-related factors** | | | | |
| Ever had terminated pregnancy | No(ref) | | | |
| | Yes | 1.22 (1.14-1.30)* | 1.09 (1.02-1.17)* | 0.006 |
| **Child-related factors** | | | | |
| Child age (in months) | ≤ 24 months(ref) | | | |
| | >24 months | 1.25 (1.19-1.31)* | 1.21 (1.15-1.27)* | 0.013 |
| Parity | ≤ 2 (ref) | | | |
| | > 2 | 1.02 (0.97-1.08) | 1.29 (1.22-1.38)* | 0.018 |

*p<0.05; $^{\mathbf{AOR}}$ Adjusted Odds Ratio; $^{\mathbf{COR}}$ Crude Odds Ratio

**Table S2.** Decomposition analysis of double burden of malnutrition

| | | | | Contribution to overall CIX=0.217 (p<0.001) | |
|---|---|---|---|---|---|
| **Factors** | | **Elasticity** | **CIX** | **Absolute contribution** | **Percentage contribution** |
| **Primary explanatory factor** | | | | | |
| Mode of delivery | Normal vaginal delivery (ref) | | | | |
| | Emergency C-section | 0.039 | 0.266 | 0.010 | 4.84 |
| | Elective C-section | 0.066 | 0.356 | 0.024 | 10.91 |
| | Subtotal | | | 0.034 | 15.75 |
| **Socio-demographic factors** | | | | | |
| Place of residence | Rural (ref) | | | | |
| | Urban | 0.068 | 0.595 | 0.041 | 18.84 |
| Advanced maternal age | No (< 35 years) (ref) | | | | |
| | Yes (≥ 35 years) | 0.034 | -0.030 | -0.001 | -0.47 |
| Maternal education | No education(ref) | | | | |
| | Primary | 0.009 | -0.269 | -0.003 | -1.21 |
| | Secondary | 0.043 | 0.128 | 0.005 | 2.55 |
| | Higher | 0.004 | 0.598 | 0.003 | 1.22 |
| | Subtotal | | | 0.006 | 2.57 |
| Wealth Index | Poorest(ref) | | | | |
| | Poorer | 0.095 | -0.391 | -0.037 | -17.13 |
| | Middle | 0.123 | 0.135 | 0.017 | 7.68 |
| | Richer | 0.134 | 0.608 | 0.081 | 37.60 |
| | Richest | 0.121 | 1.00 | 0.122 | 56.07 |
| | Subtotal | | | 0.183 | 84.23 |
| Frequency of watching television | Not at all(ref) | | | | |
| | Less than once a week | 0.007 | -0.013 | -0.0001 | -0.04 |
| | At least once a week | 0.040 | 0.506 | 0.0216 | 9.96 |
| | Subtotal | | | 0.0215 | 9.92 |
| Country | India (ref) | | | | |
| | Pakistan | 0.007 | 0.05 | 0.0004 | 0.16 |
| | Bangladesh | -0.004 | 0.06 | -0.0002 | -0.11 |
| | Maldives | 0.002 | 0.002 | -0.000 | 0.003 |
| | Nepal | -0.006 | 0.023 | -0.001 | -0.06 |
| | Subtotal | | | 0.0001 | -0.008 |
| **Maternal pregnancy-related factors** | | | | | |
| Ever had terminated pregnancy | No(ref) | | | | |
| | Yes | 0.013 | 0.05 | 0.001 | 0.29 |
| **Child-related factors** | | | | | |
| Child age in months | ≤ 24 months(ref) | | | | |
| | >24 months | 0.097 | -0.01 | -0.001 | -0.31 |
| Maternal parity | ≤ 2 (ref) | | | | |
| | > 2 | 0.075 | -0.305 | -0.023 | -10.64 |
| **Explained CIX** | | | | **0.261** | **120.18** |
| **Residual CIX** | | | | **-0.044** | **-20.18** |

CIX: Concentration Index; p = p-value

## Multicollinearity

**Stata Code:**

```
regress DBM i.modeofDelivery i.residence i.pt  i.advanced_maternal_age i.education i.wealth_index i.watching_tv i.ccountry i.child_month24 i.Parity [pw= WGT]

vif
```

**Table S3.** Variance inflation factor (VIF)

| Factors | | VIF | 1/VIF |
|---|---|---|---|
| **Primary explanatory factor** | | | |
| Mode of delivery | Normal vaginal delivery (ref) | | |
| | Emergency C-section | 1.11 | 0.90 |
| | Elective C-section | 1.08 | 0.93 |
| **Socio-demographic factors** | | | |
| Place of residence | Rural (ref) | | |
| | Urban | 1.33 | 0.75 |
| Advanced maternal age | No (< 35 years) (ref) | | |
| | Yes (≥ 35 years) | 1.08 | 0.92 |
| Maternal education | No education(ref) | | |
| | Primary | 1.47 | 0.68 |
| | Secondary | 2.23 | 0.45 |
| | Higher | 2.19 | 0.45 |
| Wealth Index | Poorest(ref) | | |
| | Poorer | 1.62 | 0.62 |
| | Middle | 1.82 | 0.55 |
| | Richer | 2.09 | 0.48 |
| | Richest | 2.45 | 0.41 |
| Frequency of watching television | Not at all(ref) | | |
| | Less than once a week | 1.45 | 0.69 |
| | At least once a week | 1.87 | 0.53 |
| Country | India (ref) | | |
| | Pakistan | 1.02 | 0.98 |
| | Bangladesh | 1.02 | 0.98 |
| | Maldives | 1.01 | 0.99 |
| | Nepal | 1.01 | 0.99 |
| **Maternal pregnancy-related factors** | | | |
| Ever had terminated pregnancy | No(ref) | | |
| | Yes | 1.01 | 0.98 |
| **Child-related factors** | | | |
| Child age in months | ≤ 24 months(ref) | | |
| | >24 months | 1.03 | 0.97 |
| Maternal parity | ≤ 2 (ref) | | |
| | > 2 | 1.27 | 0.79 |
